# New products

**DOI:** 10.1155/S1463924689000544

**Published:** 1989


Journal of Automatic Chemistry Vol. 11, No. 6 (November–December 1989), pp. 284–288

### New products

### Automated evaporation workstation

The automated TurboVap evaporation workstation can concentrate samples up to 10 times faster than Kuderna-Danish, hot block, rotary evaporation and nitrogen blowdown methods. It offers improved reproducibility and comparable recoveries. The workstation automatically and independently controls the evaporation of each sample, performs an optional solvent exchange and stops the evaporation process at a pre-set endpoint. No expensive glassware or time-consuming set-up procedures are necessary. It operates unattended and signals when it has completed. An integral water bath, which controls the thermal conditions, limits the risk of semi-volatile analyte loss. The TurboVap is compact and self-contained and can be operated on a laboratory bench. The TurboVap is available in two sizes – the 50 ml model will accommodate up to six samples (<50 ml) simultaneously; the second model accommodates up to six large samples.

*More information from the Zymark Corporation, Zymark Center, Hopkinton, Massachusetts 01748, USA. Tel.: 508 435 9500; fax: 508 435 3439.*

### Computer-based training

PERA's computer applications consultants have been developing computer-based methods of learning. Computer-Based Training is becoming popular – it provides a highly flexible training resource, which allows staff to learn from the computer at a time, location and a pace to suit themselves. A CBT package typically comprises a floppy disk containing a set of lessons and tests, and occasionally will include printed materials.

PERA's CBT experts believe that many companies gain a competitive advantage through the use of CBT as a sales and marketing tool. If a company can provide its customers with ready-made CBT packages to meet their training requirements, it is giving them the ideal resource to deal with any training requirements linked with the use of their product. This means that the CBT package provides a direct link to end users, enabling companies to develop their confidence and maintain their loyalty.

PERA suggest that accompanying products with CBT packages might result in the following advantages:

(1) Added value to products.

(2) A unique selling point.

(3) Training materials attuned to the needs of customers.

(4) Potential influence over training practices.

(5) Useful at exhibitions, trade fairs and other sales functions.

PERA's experience indicates that training is an undervalued resource from the sales and marketing perspective. Training materials can provide an excellent platform for a

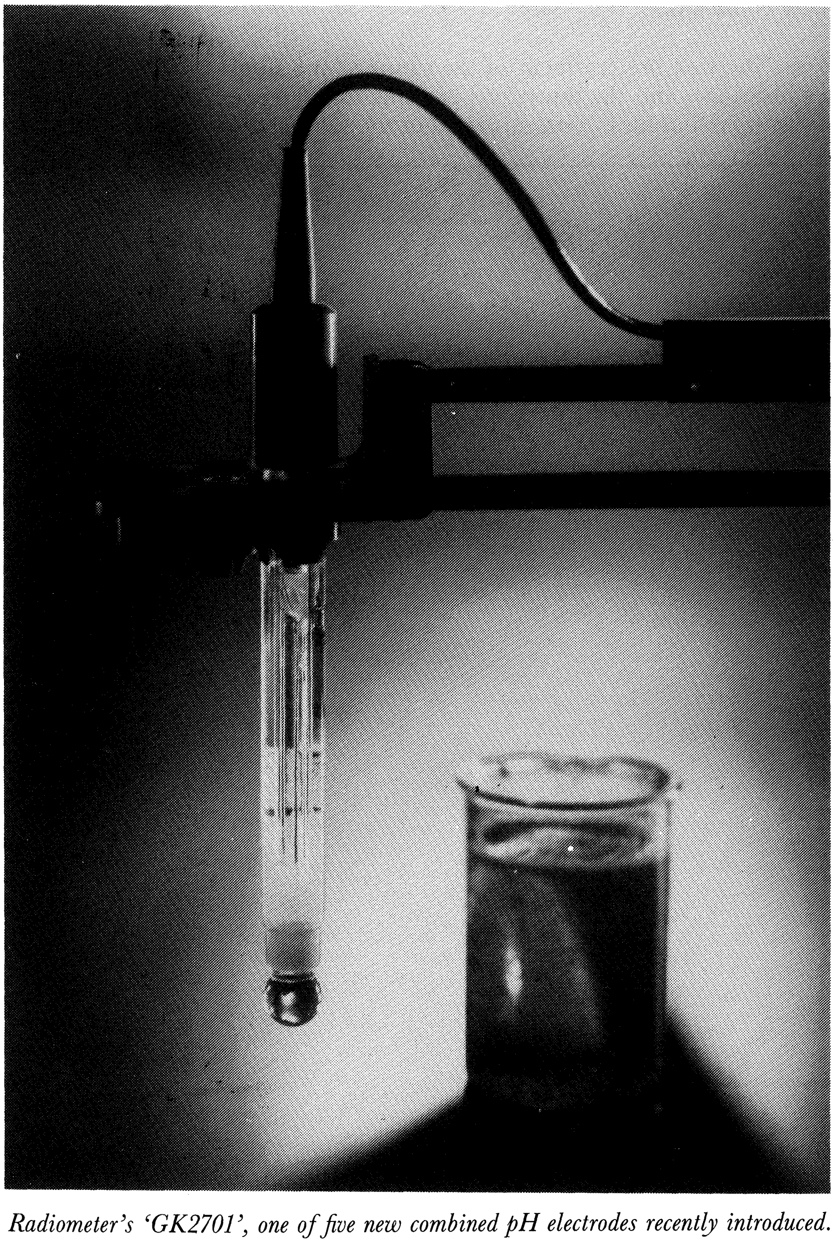

*Radiometer's 'GK2701', one of five new combined pH electrodes recently introduced.*

'soft' approach to sales, penetrating companies effectively and reaching the end users of equipment – who in turn provide feedback throughout the organization.

*For further details contact Carl Billson at PERA, Melton Mowbray, Leicestershire LE13 0PB, UK. Tel.: 0664 501501.*

### Combined pH electrode range extended

Five combined pH electrodes have been added to Radiometer's range of instruments and accessories for precise pH measurement. The first, the 'GK2701' (see illustration) is suited to routine measurements of high purity/low ionic strength water and provides excellent stability through the use of an annular ceramic junction.

The second, the 'GK2711', employs a sleeve junction to provide rapid flow of KCl filling solution. It is suited to often difficult low ionic strength solutions, colloidal suspensions, slurries and emulsions.

A spear-pointed pH-sensitive membrane suits Radiometer's third electrode, the 'GK2713', to foodstuffs like dough, meat, fish, cheese and fruit, and to such materials as leather and cosmetics.

The fourth, the 'GK2731', designed for used in soil pH measurements, as well as with blood, milk, yoghurts and brewing, has an extremely rugged construction with an annular ceramic junction providing stability.

The fifth combined pH electrode, a rugged plastic-bodied version (GK2725) should be appealing to trainers and teachers. This is also designed for long life in field use in a wide range of applications such as aqueous solutions like hops/beer musts, sea water or chlorinated swimming pools, or even milk.

*Details from Jo Kennedy, Analytical Division, Radiometer Ltd, The Manor, Manor Royal, Crawley, West Sussex RH10 2PY, U.K. Tel.: 0293 517599.*

### Cost-effective industrial monitoring

Chessell Ltd has recently announced an upgraded version of the Series 4500 Data Acquisition System. Described as a 'Building Block' distributed industrial monitoring system, the 4500 now has the ability to archive onto a series of disk drive units. This feature is ideal for users requiring historical analysis and batch quality control facilities, and further enhances the overall flexibility and tremendous expansion capabilities of the system.

The 4500 comprises a hardware and software kit which enables the user to tailor the system acording to application requirements. Designed for easy expansion, the system handles up to 885 analogue or digital, input or output channels, and scans up to 400 points in 2 s.

For cost effective, plant-wide monitoring, the 4500 is unbeatable, employing sub-systems which can be connected via a multi-drop RS422 link to allow distribution up to 1200 m. Readings may be logged to a variety of outputs, including operator interface, standard printer, analogue instrumentation – the new 4510 disk drive units – and Chessell 4001 multi-point recorders, for trending. All information can be viewed on a rugged remote operator panel, directly output by a report generator and retransmitted in analogue form. The system may also be used as an intelligent front end to host computers. Alarm scanning, annunciation and reporting are all built in.

Aimed at problem solving in the process industries, the versatile 4500 provides an economic solution to the precise measurement and output requirements of the individual user.

*Enquiries to Chessell Ltd; tel.: 0903 205222.*

### 'Victrex'

'Victrex' polyetheretherketone (PEEK) from ICI has been shown to extend the service life of pumps used in the chemicals industry. A team of materials specialists at the ICI Rozenburg site in The Netherlands have been working with pump manufacturer Begemann, ICI Advanced Materials and the Dutch Government to develop a pump which has a much longer life than those currently used in chemicals plants.

The pump development programme began at the Rozenburg MDI plant where valves lined with 'Victrex' PEEK were tested. These valves performed surprisingly well. In a very corrosive medium with high temperature the 'Victrex' lined valves were still in excellent condition after six months. The diffusion through the material was very low. Following these the team began working with Begemann to make those pump parts which are in contact with the product – the pressure cover, housing and impeller – from 'Victrex' PEEK.

For the first trials Begemann manufactured the impeller from an unfilled grade of 'Victrex' PEEK. Parts were then injection moulded from commercially available glass and carbon fibre reinforced grades of 'Victrex': impellers made from the two materials were then installed in pumps on the polyurethanes plant. Both impellers performed extremely well during service with the one made from carbon reinforced 'Victrex' showing the better performance, probably due to the higher thermal conductivity of the carbon fibre. Both impellers are still performing well after 18 months' service, while the previously used glass fibre reinforced expoxy impellers has an average life of two months.

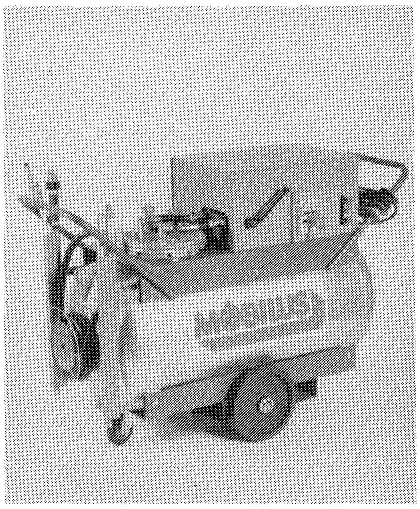

*With its achievable vacuum of 99%, 'Mobilus' trucks can draw off a wide range of materials, including highly viscous products.*

Both Begemann and ICI are confident that the new pumps incorporating 'Victrex' PEEK have a longer life than their predecessors and that they meet the stringent safety and quality standards required by the chemicals industry.

They have no doubt that the higher temperature resistance of 'Victrex' PEEK will open up a new market for non-metallic chemically resistant pumps.

*For further information contact Madeline Whitfield, ICI Advanced Materials, PO Box 6, Shire Park, Bessemer Road, Welwyn Garden City, Hertfordshire AL7 1HD, UK. Tel.: 0707 337362; fax: 0707 335556.*

### Taking care of hazardous materials

Argument rages around the best way to dispose of dangerous materials, and the discussion will increase when the UK Government's proposed 'Green' Bill becomes law. Will incineration prove to be the best solution, or landfill, or burial in seabed containers? However efficient the chosen solution, the most effective form of disposal will fail if there is spillage of the hazardous materials in transit to the disposal point. The RIETBERG system for the storage and transport of hazardous materials, now available in the UK from sole distributors EGB Industrial Supplies Ltd, could provide the answer to the problem.

It comprises a comprehensive range of tanks, storage containers, transportable containers and mobile units, all designed in accordance with stringent DIN standards and conforming to the latest International Regulations. These are the European Accord Relating to the International Transport of Dangerous Goods by Road (ADR) and the International Regulations Concerning the Transport of Dangerous Goods (RID).

Typical of RIETBERG products which are specially relevant in this situation are 'Mobilus' trucks, designed for replenishment and waste disposal duties, the trucks will handle a wide range of industrial waste products, from cutting, engine, hydraulic and gear oils to washing, cooling and cleaning fluids, as well as many other potentially dangerous products used by industry. Suitable for all types and sizes of industrial user, the trucks have a capacity of 250 l, are of compact design and weigh only 230 kg when empty.

Special attention has been paid to safety when filling and emptying the containers, which operate on the suction/pressure principle. Vacuum

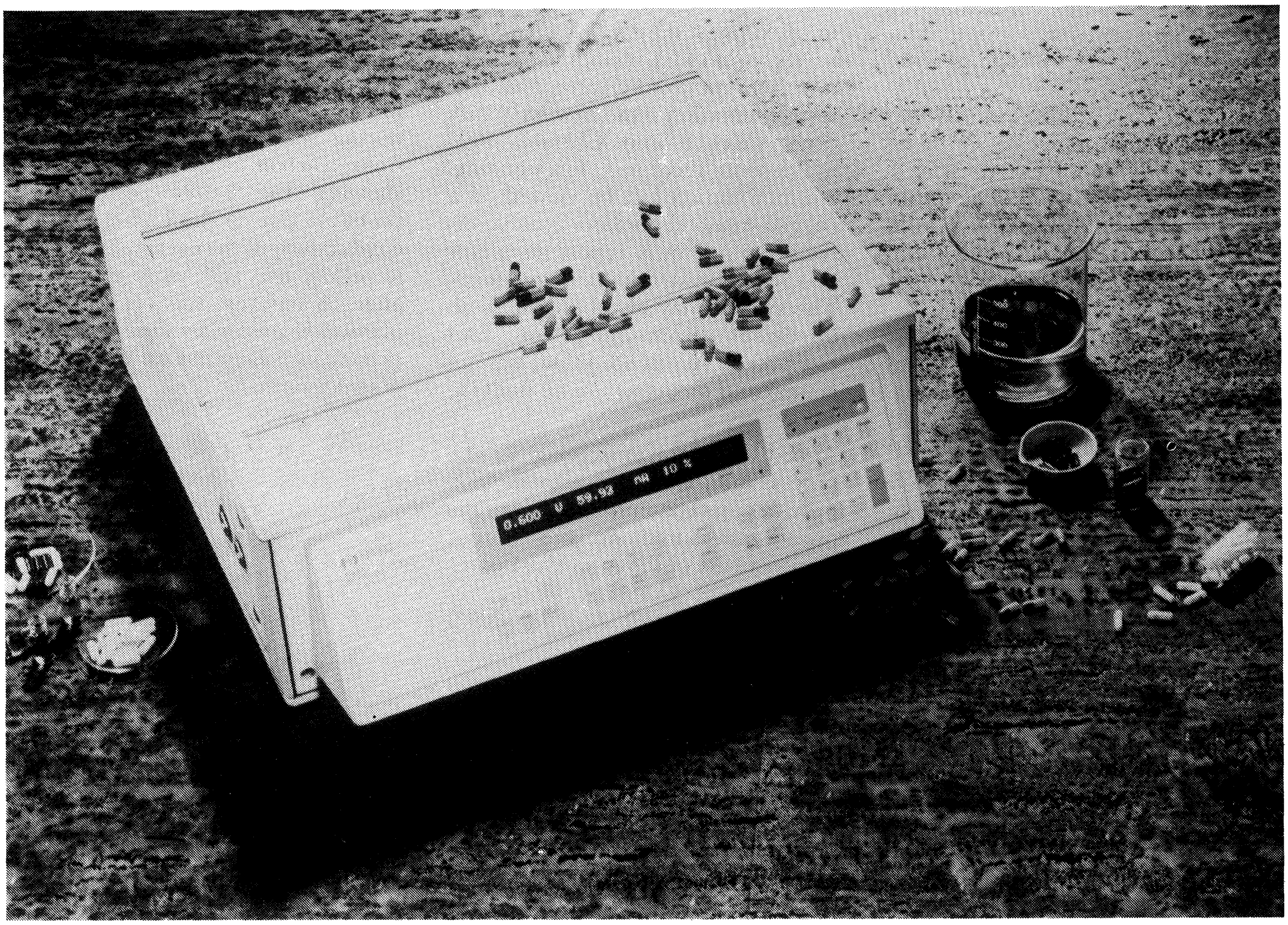

*Hewlett-Packard has introduced a new addition to its range of HPLC detectors. The HP 1049A programmable electrochemical detector can be used for analyses that are normally a problem for electrochemical detection, such as carbohydrates and catecholamines. The main application areas are pharmaceutical, clinical, environmental and food laboratories. Details from Verena Haller, Hewlett-Packard SA, 150 Route du Nant-Avril, CH 1217 Meyrin (GE) 2, Switzerland.*

is created by a quiet-running, robust sliding-valve pump and emptying is by suction lance and hose adaptor.

Both suction and decanting procedures are leak-free.

*For further information contact Colin Levene, EGB Industrial Supplies Ltd, 243–245 Horn Lane, London W3 9ED. Tel.: 01 993 2201; fax: 01 992 9632.*

### Stirrer

A new model has now been introduced to complement the existing range of IKA Magnetic Stirrers available from Sartorius. The IKAMAG RET-GS is the most advanced model, incorporating a particularly high degree of safety, advanced engineering and extremely long service life. All-round sealing prevents penetration of damaging liquids and vapours, and excellent corrosion proofing enables it to withstand rough laboratory operation.

The Magnetic Stirrer has a reliable motor with the added advantage of incorporating a 'gentle start-up' circuit. It has a 600 W silumin heating plate which is adjustable from room temperature to 300 °C and the common problem of overshooting heating plate temperature is eliminated by means of a timed proportional controller. A further safety aspect is a special electronic heating circuit which switches off the heating in the event of component failure.

The IKAMAG RET-GS is available with a range of optional accessories including sets of stirring bars, an extension plate, support rod, meter, inserts for round flasks, beakers and test-tubes and safety contact thermometer with connecting cable.

*For further information contact Peter Butler, Sartorius Ltd, Longmead Industrial Estate, Blenheim Road, Epsom, Surrey KT19 9QN, UK. Tel.: 03727 45811.*

### HP LAB/UX, a flexible, UNIX-based LIMS

Analytical laboratory productivity is much increased with Hewlett-Packard's new HP LAB/UX laboratory information management system (LIMS). UNIX based, the new system consists of proven LIMS software, supports industry standards such as the X Window System, and allows access to standard networking technologies and electronic mail. The system is flexible, and can be configured to individual laboratory requirements without re-programming. Data security and validation procedures help meet Good Laboratory Practice (GLP) requirements.

*Details from Verena Haller, Hewlett-Packard (see below).*

### Lab automation software

Hewlett-Packard has introduced the first major software revision for the HP 3359A Chromatographic Worksystem. The new software offers several improvements to this laboratory automation system, providing a multiple instrument, multi-user package for high-volume, routine laboratory analyses. The system increases laboratory productivity for both gas and liquid chromatography.

Data storage capacity is increased; an automated disk management facility automatically purges selected, infrequently used files to prevent the accumulation of unwanted data. The newly supported 304-megabyte HP 7959 disk drive doubles system storage capacity and provides faster storage and access of data.

Improvements to the integrator provide faster report generation

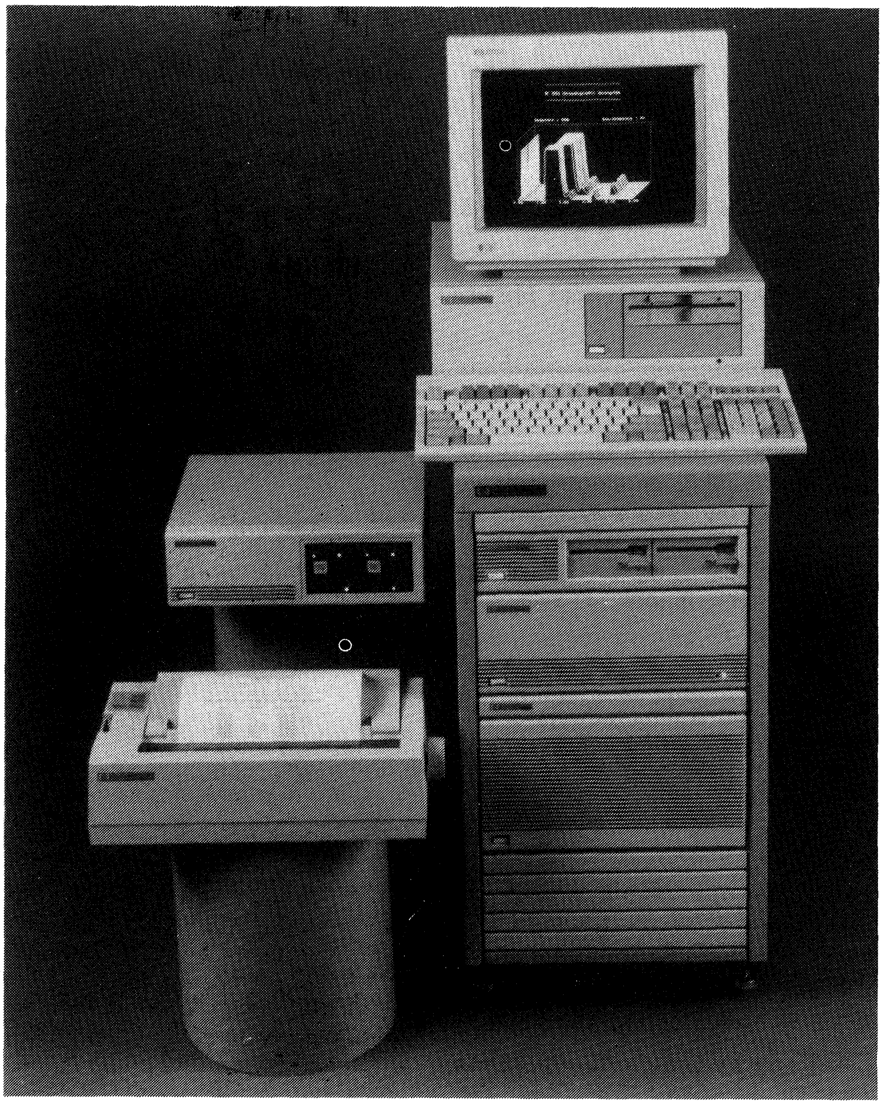

*The HP 3359A chromatographic worksystem from Hewlett-Packard Company and its new software increase laboratory efficiency for high-volume, routine gas- and liquid-chromatographic analyses.*

and chromatographic integration. During manual reprocessing of analytical data, the running status is displayed, allowing the operator to track the progress of the analysis.

The new software supports additional HP applications on the HP 3359A Chromatographic Worksystem. For efficient fractionation of petroleum products, simulated distillation by gas chromatography determines boiling-point distribution of unknown samples. HP CLP Plus software simplifies and speeds up the entire process of analysing, reviewing and reporting total organics data.

An optional Telnet package improves networking capabilities. As well as the existing facility to transfer data and files to and from the HP 3359A, users can now access other HP and non-HP computers via a local area network. In turn, other computers can access the HP 3359A.

*Enquiries to Verena Haller, Hewlett-Packard SA, 150 Route du Nant-d'Avril, CH 1217 Meyrin (GE) 2, Switzerland.*

### Sourcebook for analytical HPLC columns

Over the years, *Waters's Sourcebook* has become much more than a brochure showing lists of available HPLC columns. It is important reading for the professional chromatographer, with advice on column selection and a detailed appendix covering the factors affecting column efficiency and the performance of difference bonded phases.

Waters's 1990 price list, which accompanies the Sourcebook, includes the following new launch:

The complete range of SHODEX columns and chemistries which have significantly enhanced the company's capability in the analysis of sugars and carbohydrates, high performance affinity LC, high temperature GPC and the isolation/purification of proteins.

*Copies from Waters Chromatography Division, Millipore (UK) Ltd, The Boulevard, Ascot Road, Croxley Green, Watford WD1 8YW, UK. Tel.: 0923 816375; fax 0923 818297.*

### Analytical weighing

The AT201 semimicro analytic balance has a 205 g weighing range with a continuous readability of 0·01 mg. This corresponds to a resolution of 20 million points (weighing range divided by readability). This high precision is assured by FACT (Fully Automatic Calibration Technology). An additional inner draft shield shields the weighing pan from disturbing air turbulence. This balance is especially suitable for the determination of extremely small weight differences of a relatively heavy sample.

The AT201 has all the technical benefits of the recently introduced AT series, for example the dynamic METTLER DeltaTrac graphic indicator and a fully automatic draft shield.

Mettler is also marketing the AT261 with DeltaRange. With this analytical balance the user has, in effect, two balances in one: a classic analytical balance in the macro range and a semimicro balance which can be called up via the DeltaRange. The AT261 is particularly suitable for the weighing of small quantities into heavy vessels, as well as for very precise formula weighings over a wide weight range. The semimicro range can be called up at any point over the entire weighing range of 205 g (readability 0·1 mg). This DeltaRange always comprises a weighing range of 62 g with a readability of 0·01 mg.

The new AT balances are equipped with a current loop and an RS232C interface as standard.

*Details from Mettler Instrumente AG, CH 8606 Greifensee, Switzerland.*

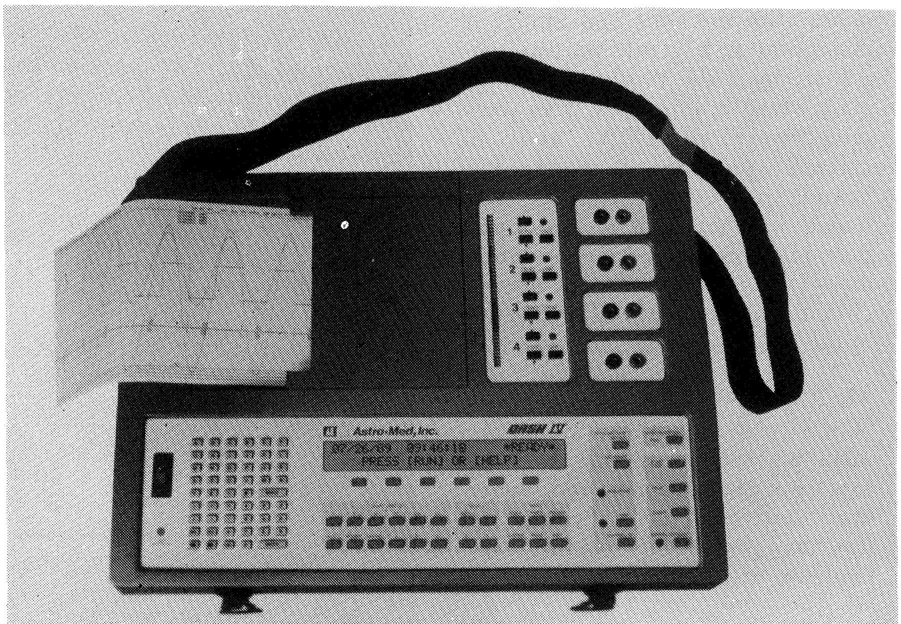

*A compact, lightweight four-channel field and laboratory recorder featuring extraordinary capabilities and complete reliability has been announced by Astro-Med, Inc. The recorder (Dash IV) offers real-time frequency response from DC to 25 kHz full-scale; internal rechargeable battery, AC and 12 VDC operation; on-demand self-calibration; and data capture and playback with a capture memory of 64 K samples per channel. The unit was designed to operate reliably in adverse environments, for example steel and paper mills, and nuclear power stations. The Dash IV offers simple operation – menus displayed on an LCD guide the user through the entire programming sequence. Waveforms are extremely sharp, and accuracy is assured by the self-calibration feature of the Dash IV. Full details from Astro-Med, Inc., Astro-Med House, 11 Whittle Parkway, Slough SL1 6DQ, UK. Tel.: 0628 668836.*

